# Intimate partner violence among HIV index case testing clients in Malawi, a mixed-methods study

**DOI:** 10.1371/journal.pgph.0003818

**Published:** 2025-02-25

**Authors:** Maria Sanena, Christine Hagstrom, Khumbo Phiri, Pericles Kalande, John Songo, Linda Maruwo, Sam Phiri, Kathryn Dovel, Joep J. van Oosterhout

**Affiliations:** 1 Partners in Hope, Lilongwe, Malawi; 2 Department of Medicine, David Geffen School of Medicine, University of California Los Angeles, Los Angeles, United States of America; 3 School of Global and Public Health, Kamuzu University of Health Sciences, Lilongwe, Malawi; University of Manitoba College of Medicine: University of Manitoba Max Rady College of Medicine, CANADA

## Abstract

While its prevalence in sub-Saharan Africa is high, intimate partner violence (IPV) is rarely reported during routine index case testing (ICT) services in Malawi. We retrospectively explored the occurrence of IPV in a large, PEPFAR-supported HIV care and treatment program, among people with HIV (PWH) who had previously accessed ICT services. Between July-August 2022, we enrolled PWH aged ≥18 years, at 15 health facilities, who had received ICT services as new ART-initiators <6 months ago. We used a validated World Health Organization tool to measure IPV in domains of physical, sexual and emotional abuse, and controlling behaviors in the past 12 months. To understand reasons for IPV non-reporting, we randomly selected a sub-set of PWH who had experienced IPV and healthcare workers (HCWs) providing ICT services, for in-depth interviews (IDIs). We enrolled 149 PWH, 71.8% were female, 58.4% married, mean age 34 (IQR 25-40) years. Overall IPV prevalence was 71.1% (95%CI: 63.8%-78.5%), 76.6% (95%CI 68.5%-84.8%) among females and 57.1% (95%CI: 41.5%-72.8%) among males. Controlling behavior (65.7%) was the most common form of IPV, followed by physical (24.8%), emotional (33.6%), and sexual abuse (20.8%). Twenty-two percent related the IPV event to the HIV diagnosis. We analyzed 24 IDIs (15 PWH, 9 HCWs). Only two PWH (13.3%) had reported IPV to a HCW. Major barriers to reporting IPV for PWH were believing it is inappropriate discussing IPV with HCWs, concerns that discussing IPV would cause delays for others, and limited privacy. HCWs attributed IPV non-reporting to insufficient screening, high workload among ICT staff, negative HCW attitudes, and PWH’s fear of unintended consequences of IPV reporting. IPV prevalence was high among PWH who had recently undergone ICT, but IPV is often not reported. Multiple barriers to IPV reporting must be addressed urgently, given IPV’s adverse impact on HIV treatment and other health outcomes.

## Introduction

Intimate partner violence (IPV) is an important public health issue worldwide [1], including in sub-Saharan Africa where approximately 44% of women aged 15-49 have experienced some form of IPV [2]. Although data is more limited, men also experience IPV; approximately, a quarter of married or cohabitating men in Sierra Leonne and Cameroon reported having faced IPV [3]. The prevalence of IPV is higher among people with HIV (PWH) when compared to HIV-negative populations [4], and IPV is associated with poor ART adherence [5]. Further, a study of PWH in Uganda found that 12.2% of people experiencing IPV reported that it was related to their partner learning about their HIV positive status [6].

Index Case Testing (ICT) is an evidence-based strategy to increase the number of PWH who know their status [7]: after a new case is identified, health facility staff interview the client about their sexual partners and biological children, and offer passive and active strategies for testing these exposed contacts. Despite its success as a case finding strategy, ICT may increase the risk of IPV upon partner notification [8]. To reduce ICT-associated IPV, screening for IPV risk and referrals to IPV support services have become part of standard operating procedures for ICT. Depending on the level of risk, the client and provider may opt for anonymous partner notification, notification with support of a health care worker present, or may choose to forgo partner notification entirely to ensure no harm comes to index clients, partners, or family members [9].

Following Malawi Ministry of Health (MOH) guidelines [10], Partners in Hope (PIH), a Malawian non-governmental, medical organization and PEPFAR clinical implementing partner, began implementing IPV screening and risk assessment among ICT clients in 2019. However, up to 2022, not a single case of IPV associated with ICT was reported, which is in stark contrast to local IPV prevalence data, indicating that in Malawi, approximately a quarter of women aged 15-49 report experiencing IPV recently [11]. We therefore conducted a cross-sectional survey using a mixed methods approach to explore the occurrence of IPV among PWH who had accessed ICT, despite non-reporting within routine program implementation. We also aimed to identify reasons for non-reporting of IPV cases and to identify barriers to IPV screening, monitoring, and reporting by interviewing PWH and health care workers (HCWs). Based on the study’s findings, we planned developing interventions to improve IPV reporting.

## Methods

### Setting

According to World Bank criteria, Malawi is the 4^th^ poorest country in the world. The total health care expenditure per capita is only 40 USD per annum. Malawi has a high HIV prevalence among adults (15-49 years) of 7.1% (women 8.5% and men 5.2%), with close to 13,000 new adult infections per year [11]. Despite poverty and high HIV burden, Malawi has a successful HIV program: 94% of PLHIV know their HIV status, 93% of those are on anti-retroviral therapy (ART), with 87% viral load suppression [11]. The national HIV program started assisted partner notification (active ICT) in 2019, targeting selected index clients, i.e., individuals newly diagnosed with HIV, new ART initiates, and ART clients with a new high viral load result, whose sexual partners and/or biological children had unknown HIV status. An IPV risk assessment, conducted by HIV Diagnostic Assistants (HDAs), a lay cadre staff implementing HIV testing services, was routinely included in ICT screening.

### Survey

From 1 July through 31 August 2022, PIH conducted a survey of PWH who had received ICT services at 15 high volume ART clinics (5 each in Lilongwe, Mulanje, and Chikwawa districts). Participants were selected from ICT registers, enrolled into the survey and interviewed about IPV during routine ART clinic visits. Three enrollment criteria needed to be present: age at least 18 years; having been screened for ICT not more than 6 months earlier; and having had one or more sexual partners with unknown HIV status in the last 12 months. All eligible PWH who visited the ART clinics in the study period were enrolled. The occurrence of IPV was measured using a World Health Organization-validated survey on gender-based violence [12]. The survey tool assesses occurrence of IPV in 4 main domains: physically violent acts, sexually violent acts, emotional aggression, and controlling behaviors. IPV was present if a participant responded yes to any question in any of the four domains. Participants were also asked if they felt that the occurrence of an IPV act was related to their HIV status. We used descriptive analyses to describe the prevalence of IPV within each domain and across the subcategories of socio-demographic characteristics. Data cleaning and statistical analyses were conducted using Stata 18.0. (StataCorp LLC, Texas, USA)

### In-depth interviews

We conducted in-depth interviews with 15 PWH and 9 HCWs from three health facilities in the last two weeks of August 2022. PWH who screened positive for IPV in the survey were eligible for inclusion in the in-depth interviews and five per health facility were randomly selected. In-depth interviews focused on PWH’s experience with IPV, if they had ever reported it to a health care provider and if not, any reasons for non-reporting, including awareness of places where IPV can be reported. We also took a purposive sample of 9 HCWs, including clinical officers, nurses, HDAs, Treatment Supporters (TS), and data clerks. In the in-depth interviews with HCWs, we asked what prompts HCWs’ suspicion that a client has experienced IPV, which factors facilitate or impede HCWs to do routine IPV screening, which recommendations they have to increase HCWs’ identification of IPV during HIV care appointments, the availability of IPV screening tools, and to what extent guidelines on IPV screening and IPV referrals are implemented. In-depth interviews were audio recorded and analyzed by the study team. The audio recordings were transcribed, translated to English, and imported to Atlas.ti software (Atlas.ti version 8; Scientific Software Development GmbH, Berlin, Germany) for coding and analysis. A codebook was developed focusing on demographics, IPV screening experience, referral and follow-up practices, and health system-related issues regarding IPV case management. The codebook was piloted and revised as needed by three members of the study team prior to analysis. We used summary statistics for demographic data and deductive and inductive approaches to analyze thematic areas of qualitative data.

### Ethics statement

We received ethics approval from the Malawi National Health Sciences Research Committee, protocol number 1099. We obtained and documented verbal informed consent from HCWs (for the in-depth interviews) and clients (survey and in-depth interviews). We provided a Malawi Kwacha equivalent of USD 5 to study participants, following national standards for compensation of research participation at the time.

## Results

### PWH Surveys

149 adult PWH who had undergone ICT and were since receiving ART services, were enrolled in the survey. The proportion of females was 71.8% and 58.4% were married. 38.9% had been contacted in their community for follow-up by an ICT service provider and a large majority (83.2%) had disclosed their HIV status to their partner. Among participants, males were more often married or cohabiting with a partner, were more often employed, had higher earnings and had higher education levels (Table 1).

**Table 1:** Characteristics of Malawian adults who had undergone ICT (n=149).

	Total (n=149)		Male (n=42)		Female (n=107)		
	N	%	N	%	N	%	p-value
***Marital status***							
Single	11	7.4%	3	7.1%	8	7.5%	.008
Cohabitating/Partnered	21	14.1%	8	19.1%	13	12.1%	
Married	87	58.4%	30	71.4%	57	53.3%	
Widowed/Divorced/Separated	30	20.1%	1	2.4%	29	27.1%	
***Employment***							
Employed (full or part time)	80	53.7%	28	66.7%	52	48.6%	.047
Not employed	69	46.3%	14	33.3%	55	51.4%	
***Education***							
Primary	37	24.8%	8	19.1%	29	27.1%	.047
Secondary/Higher	17	11.4%	9	21.4%	8	7.5%	
No school/did not complete primary	95	63.8%	25	59.5%	70	65.4%	
***Earnings/Income***							
Able to save	10	6.7%	6	14.3%	4	3.7%	.003
Only enough to meet expenses	71	47.7%	25	59.5%	46	43.0%	
Not sufficient	68	45.6%	11	26.2%	57	53.3%	
***Urban/Rural***							
Urban	21	14.1%	7	16.7%	14	13.1%	.572
Rural	128	85.9%	35	83.3%	93	86.9%	
***District***							
Lilongwe	53	35.6%	14	33.3%	39	36.5%	.790
Chikwawa	49	32.9%	13	31.0%	36	33.6%	
Mulanje	47	31.5%	15	35.7%	32	29.9%	
***Ever followed up for ICT***							
No	91	61.1%	29	69.1%	62	57.9%	.211
Yes	58	38.9%	13	31.0%	45	42.1%	
***HIV status disclosure to partner***							
No	25	16.8%	4	9.5%	21	19.6%	.138
Yes	124	83.2%	38	90.5%	86	80.4%	

Among PWH, 71.1% (106/149) reported experiencing IPV in the past 12 months. IPV prevalence was 76.6% among women vs. 57.1% among men (p= 0.018). The most common type of IPV was controlling behavior (65.8%), followed by emotional abuse (33.6%), physical abuse (24.8%), and sexual abuse (20.8%). All four subcategories of IPV were significantly more commonly reported by women (Table 2). Of the 106 clients who reported an IPV experience in the previous year, 21.7% felt that the IPV was due to their HIV diagnosis, which was 24.4% among women vs. 12.5% among men (p= 0.214). Anonymized data of PWH surveys are available as supplementary materials (S1 Data).

**Table 2:** Prevalence of IPV and IPV subcategories among adult PWH who underwent ICT screening in the past 6 months.

	Total (n=149)		Male (n=42)		Female (n=107)		
**IPV domain**	**N**	**%**	**N**	**%**	**N**	**%**	**p-value**
Emotional abuse	50	33.6%	9	21.4%	41	38.3%	.049
Physical abuse	37	24.8%	4	9.5%	33	30.8%	.007
Sexual abuse	31	20.8%	0	N/A	31	29.0%	<.001
Controlling behavior	98	65.8%	20	47.6%	78	72.9%	.003
Overall	106	71.1%	24	57.1%	82	76.6%	.018

### PWH In-depth Interviews

We conducted in-depth interviews with 15 PWH who had reported IPV in the survey. Twelve were female, median age was 30 years, and 10 lived with their partner. When asked if they had ever reported or shared their IPV experiences with anyone, only two PWH (13.3%) mentioned sharing their IPV experience with a health care provider, but all said that they had told someone, usually a relative, about their experience with an abusive partner. PWH cited several reasons for not reporting IPV at a health facility. Some indicated that they only started experiencing IPV after they were diagnosed with HIV and had disclosed their status to their partners, however, most reported experiencing already IPV for a longer period. Some mentioned that they did not know they could share relationship issues or experiences of IPV with their health care provider, and others either didn’t feel comfortable sharing the IPV experience or didn’t feel that it was the appropriate place to discuss such issues. PWH felt that discussing IPV was better suited to a marriage counsellor or a family member.

“Its a place where we can just go to get the drugs not as in marriage counselors, I just think that only marriage counselors are the ones to help us.” – Male, age 50

Also, PWH who were newly diagnosed with HIV indicated a desire to leave the health facility quickly, in order to process their own feelings about their HIV diagnosis and out of fear that other clients might assume that they had tested positive if their appointment lasted too long. Further, they didn’t feel it was fair to clients waiting in the queue if they took too much time with the provider.

“There is usually a long queue at the facility waiting to see the doctor and for me to hold the queue because I am complaining about my marital issues is unfair to others.” – Female, age 33

PWH all valued privacy when it comes to issues of IPV, and they highlighted that limited private space at health facilities made them hesitant to disclose IPV for fear of being overheard. Some clients also expressed concern that providers may not maintain confidentiality if they were to disclose.

“Sometimes I am afraid that if I tell the HCW my problems, will they not go out and just tell everyone about it?” – Female, age 41

PWH agreed they would feel more comfortable opening up about IPV if their health care provider showed genuine interest in them, was approachable and friendly, and reassured them about confidentiality.

#### *Provider* in-depth interviews.

We conducted 9 in-depth interviews with HCWs. Their median age was 30 years and they were in their current role for a median of 2.5 years. Eight mentioned that they had seen clients with IPV. HCWs agreed that lack of privacy and negative attitudes from HCWs prevents clients from opening up about IPV. Nearly all HCWs stated that IPV screening does not take place consistently at facilities, primarily due to a heavy workload of HDAs and a high volume of clients. They indicated that HDAs’ main focus is on HIV case identification, and given time constraints, the quality of counselling they provide suffers, making IPV screening less thorough and consistent.

“Workload and long queues with other clients shouting/complaining that it is taking long, we end up having pressure and we don’t spend much time with the clients” – female HDA

HCWs thought that fear of unknown or unintended consequences of disclosing (e.g., the partner getting arrested) may prohibit clients from sharing IPV experiences. They also thought that given gender- and cultural norms, it is possible that clients don’t recognize their experiences as IPV, particularly regarding emotional abuse and controlling behaviors. To increase IPV case identification and reporting, HCWs suggested several interventions: hanging posters featuring standard operating procedures on IPV screening in HIV service rooms of health facilities as a reminder for HDAs; offering training to HDAs to improve interpersonal communication skills, and assuring clients about confidentiality regarding IPV. Barriers to reporting IPV, as mentioned by PWH and HCWs in IDIs, are summarized in Table 3.

**Table 3:** Summary of perspectives of people with HIV and health care workers on barriers and facilitators to reporting IPV at a health facility.

	Barriers	Facilitators
**PWH**	Felt uncomfortable or that IPV was inappropriate to discuss Concerned about spending too much time at the facility Lack of privacy and confidentiality at facility	A sense that providers care about them Friendly, approachable provider demeanor Reassurance about confidentiality
**HCWs**	Poor quality of screening Inconsistent screening Competing priorities and time constraints Lack of privacy at facility Negative HCW attitudes Client fears of unintended consequences	• Improved interpersonal skills through training providers • Reassuring clients about confidentiality • Reminders for providers to screen (posters with standard operating procedures of IPV screening)

### Interventions

After analyses of the survey and IDIs, and in line with updated PEPFAR recommendations, we organized HCW re-orientations on IPV and introduced dedicated IPV registers at health facilities. Staff started reporting clinically suspected IPV cases consistently in IPV registers and documented referrals to post-GBV service stations. Between October and December 2023, 449 IPV cases were registered at the 15 health facilities of the survey, of whom 217 cases were on ART, 30 had been newly HIV diagnosed, 130 were HIV negative and 77 had an unknown HIV status. Specific data on whether IPV was associated with ICT are still not available, given that guidelines do not recommend routine IPV screening of clients after the ICT process. While the interventions improved IPV reporting, it remains unknown whether the ICT process was the catalyst for the reported IPV.

## Discussion

Our findings indicate that a high percentage of PWH, who had undergone ICT up to 6 months earlier, had experienced IPV (71.1%), despite the fact that no IPV had been reported at the same health facilities in routine practice. The IPV prevalence was higher among females and around one in five participants related the IPV experience to their HIV diagnosis. The most commonly reported types of IPV were controlling behavior and emotional abuse. Physical and sexual abuse were each reported by around one in three participants and were significantly more common among females. ICT clients and HCWs provided detailed perspectives on IPV reporting barriers and facilitators that were related to stigma, privacy and confidentiality, HCW attitudes and skills, and the high HIV service burden at clinics.

In a large retrospective survey from Cameroon, based on programmatic active ICT data from between 2007 and 2015, IPV was reported infrequently (1.1%). However this survey only reported “physical IPV” and may not have used the validated WHO tool to determine IPV [13]. A cluster randomized trial of active ICT in Kenya documented much lower percentages of each of the IPV components than in our survey, but the overall prevalence of IPV was not indicated [14]. A systematic review from 2017 that included >5,000 ICT clients from 10 studies, reported that the prevalence of social harms attributable to active ICT was low (0-3%) but did not summarize the overall occurance of IPV among ICT clients [15]. In a recent trial of HIV self-testing added to passive ICT from Malawi, a low overall prevalence of IPV was observed (<3%) [16]. Reasons why we detected a much higher prevalence of IPV than in these studies may include the specific focus on IPV of our survey, which may have negated some barriers to routine IPV reporting; the use of a validated, sensitive diagnostic tool; a higher threshold to exclude individuals with IPV risk and the fact that we asked participants to report IPV over a longer period. The prevalence of IPV was also substantially higher than the WHO’s estimate of lifetime IPV prevalence among ever-partnered girls and women aged 15-49 in Malawi, irrespective of HIV status, which is 38% [17]. This underlines research indicating that PWH experience IPV at higher rates than the general population [4], especially around the time of HIV diagnosis.

Findings from IDIs with PWH and HCWs indicated facilitators and barriers to IPV reporting. PWH emphasized having discomfort about discussing IPV with HCWs. Reasons for that discomfort that may be relatively easily addressed include the sense that a health facility is not the appropriate place for clients to discuss IPV issues, concerns about spending too much time for a consultation and thereby hindering other clients, poor attitudes of HCWs, insufficient HCW communication and counseling skills, and concerns that HCWs may not guard confidentiality sufficiently. Health education and demand creation for IPV management as a priority health care intervention may help clients overcoming reservations on IPV reporting. Intensive HCW orientation and training on IPV can address current professional gaps regarding IPV management among HIV service providers and other HCWs as well as making them more emotionally supportive [18].

Echoing previous research [18–22], participants in our survey complained about the lack of privacy at health facilities, leading to understandable worries about being overheard and creating a strong hinderance to disclosing IPV to HCWs. Due to longstanding underfinancing of the health care sector [23,24], there is inadequate infrastructure at many governmental health facilities. Lack of privacy is therefore a common reality that may not be addressed soon. Another negative factor is the fear of unintended consequences of IPV reporting (e.g., further violence or ending of a marriage/relationship, resulting in financial harm for women in particular). Apart from the availability of ad hoc IPV survival management, fully addressing these fears will require structural, cultural changes at a population level that may take longer to achieve. Despite these barriers, evidence suggests that many women want HCWs to ask them about domestic violence [25,26], and PWH who are probed about IPV report higher satisfaction with care [26]. Therefore, HCWs should provide targeted screening for IPV, which may be aided by simple health system strengthening measures such as making dedicated monitoring and evaluation tools available and providing job aids and posters with instructions for IPV screening and management. In our own setting we have seen that this led to improved reporting of IPV and documentation of referrals for IPV management.

We note some limitations of our study. The survey and in-depth interviews took place up to 6 months after ICT and our survey did not specifically enquire about a specific relationship between IPV and the ICT process. The WHO gender-based violence tool that we used, does not report economic abuse as a specific form of IPV, while it may be common in our setting, particularly in women [27]. We observed the association between IPV and a new HIV diagnosis, but cannot conclude from the survey or from data collected after implementation of IPV reporting improvements, if the observed IPV was directly related to ICT. Survey questionairres were taken by research assistants who were not involved in health care delivery, but desirability bias may have impacted some findings. Our study took place at high-burden health facilities only and findings from the survey and in-depth interviews may not be representative of smaller and more rural settings in Malawi.

## Conclusions

While no IPV had been reported in programmatic settings, we found that the prevalence of IPV among ICT clients was high, with a particularly high burden of physical and sexual violence, especially among women. One in five participants related IPV to their HIV diagnosis but a direct relationship to ICT could not be established as such information is not collected. Barriers of IPV reporting at health facilities, indicated by ICT clients and HCWs, included stigma, lack of privacy and confidentiality, poor HCW attitudes, inadequate HCW skills and the high HIV service burden at clinics. Several interventions can address “low hanging fruit” barriers, including provision of dedicated job aids and registers, HCW training and health education for clients, while other barriers depend on factors largely rooted outside the health care sector. More research is needed to determine what kind of training, reminder systems, and other intervention strategies are efficient and effective at ensuring consistent identification and accurate capturing of IPV experiences.

## Supporting information

S1 Data Anonymized data from surveys with People with HIV. (XLSX)
